# The prognostic merit of self-reported triggers of recurrent low back pain: study protocol

**DOI:** 10.1186/s12998-019-0291-6

**Published:** 2020-01-16

**Authors:** Emad M. Ardakani, Charlotte Leboeuf-Yde, Angela Jacques, Bruce F. Walker

**Affiliations:** 1 College of Science, Health, Engineering, and Education, Murdoch University, Murdoch, 90 South St, Murdoch, Perth, Western Australia 6150; 2 Institute for Regional Health Research, University of Southern Denmark, Odense, Denmark; 3 School of Physiotherapy and Exercise Science, Curtin University, Bentley, Australia

**Keywords:** Non-specific LBP, Trigger, Trajectory, Prognosis, Prevention

## Abstract

**Background:**

Most cases of low back pain (LBP) are regarded as non-specific and current studies indicate that for many this is a chronic recurrent condition, in which people experience episodes of pain with symptom-free periods in between. It is likely that acute exposure to some factors triggers the reappearance of new episodes in recurrent LBP regardless of the causality of the underlying condition (i.e. risk factors). Additionally, it has been shown that LBP patients present with different trajectories and different trajectories possibly have different triggers. Hence, dividing patients into some clinically meaningful subgroups may offer new insights into triggers, effective preventive strategies and, therefore, prognosis. This study aims to identify self-reported triggers and trajectories of episodes of recurrent LBP and to examine the prognostic association between different triggers and LBP trajectories.

**Methods:**

This is a longitudinal, multicentre, Australia-wide observational study of patients with recurrent non-specific LBP. Two hundred adults with at least a one-year history of LBP will be recruited from primary care clinics or private practices and followed for a year. Each will receive an SMS every fortnight (26 time-points in total) enquiring the occurrence of a new episode of pain in the past 2 weeks and its intensity. Upon report of a new episode, a telephone interview will be performed to appraise exposure to self-nominated triggers in a period of 24 h preceding the pain. Trajectories will be identified by latent class analysis at the end of the follow-up based on the pain intensity, frequency, and length of episodes. Triggers will be categorised into physical and psychosocial groups. Generalised linear mixed models with logit links will be used to explore pain triggers associated with pain trajectories.

**Discussion:**

The completion of this study will provide insight into the patients’ self-reported triggers of LBP and also their possible prognostic association with different trajectories. Some newly-identified and pre-identified triggers are likely to be found and reported.

## Background

Low-back pain (LBP) is among the most common causes of disability globally with a point prevalence of about 10–30% of the world population, with pain ranging from mild to severe [1–3]. It is endemic, being reported by about 80% of people at some point in their life [4–6]. In the Global Burden of Disease study report (GBD 2017), LBP was ranked as the leading cause of disability [7]. Also, for many, LBP is a chronic lifetime condition that imposes a significant burden on them and the health budgets of many countries [8].

The economic burden of chronic LBP such as inpatient or outpatient physical therapy, pharmacy, and work absenteeism is considerable and increasing, and seems to be comparable to the cost of other chronic conditions like heart disease or diabetes [9]. There are numerous treatment options used for LBP, with almost 30% of LBP management costs allocated to physical therapy and medications [10]. However, there is still no over-arching effective treatment of choice for the type of LBP that is described as ‘non-specific’ [11]. It is so called because its aetiology is unknown and, therefore, remains a condition that is difficult to manage for both clinicians and policymakers [12, 13].

It is shown that the natural history of non-specific LBP does not follow precisely an acute, subacute or chronic pattern as described by Nachemson and Bigos back in 1984 [14]. Today, it has been noted that in the general population and primary care setting, low back pain appears to be mostly persistent or recurrent [15]. This manifests as either a persistent episode of pain with different levels of severity (i.e. flare-ups) over a long time or a new episode of pain after a period of being pain-free. Hence, despite common beliefs that LBP is a self-limiting condition, its prognosis is not as favourable as previously believed since it recurs in most instances, and many sufferers experience continuous or multiple episodes of pain in the course of the disease [16–24].

LBP, in fact, could be compared to other chronic recurrent diseases such as asthma or migraine where it is believed that the ‘disease’ needs to be established first (i.e. the onset of the very first symptoms where one was disease-free/symptom-free previously), followed by episodes of exacerbations later during the course of the disease. Likewise, some people have the ‘disease’ of LBP with many ensuing back pain episodes (exacerbations) throughout their lives [25]. Thus, it is plausible to approach LBP like asthma or migraine in which there is an underlying cause or a number of causes for the ‘disease’ (known as risk factors), while many factors contribute toward its exacerbations (acknowledged as triggers). This concept of an underlying ‘disease’ and consecutive episodes is crucial in the research of episodic, recurring conditions, particularly when risk factors (RFs) are likely to be different from triggers. Consequently, an acknowledgement of a disease-free period and an accurate, precise definition of an episode and non-episode (a pain-free break between two distinct episodes) seems to be indispensable when investigating RFs or triggers of LBP [26].

In 2002, de Vet et al. highlighted the necessity for a clear definition of an episode and non-episode in order to bring unanimity into LBP research and make the interpretation of research findings more tangible [27]. Since they could not find a unanimous definition through a review of the literature, they proposed new definitions for an episode and non-episode following group discussions. This was: “An episode of low back pain is defined as a period of pain in the lower back lasting for more than 24 hours, preceded and followed by a period of at least one month without low back pain”. The proposal was based on what was commonly used in research plus patients’ abilities to recall the pain [27]. However, a relatively recent systematic review of the definition of recurrent low back pain noted that there is still a great diversity in the definition of recurrent LBP [28]. de Vet’s definition can be broken down into two components: 1) definition of pain period (episode) and 2) definition of pain-free period (non-episode). Although the validity of the definition of a non-episode (four weeks pain-free period) was studied in the general population and primary care and shown to be applicable [29, 30], their proposed definition for an episode (a LBP for at least 24 h) has not been scientifically confirmed.

With the Acknowledgement of LBP as an episodic, recurrent disorder, studying its course by just measuring, observing, and reporting on a few episodes or a few points in time at long intervals will not likely reflect the actual long-term course of LBP since data are missed between those measurements. In other words, those discontinuous measurements will not adequately describe the pain profile over time. In this respect, some studies have investigated LBP trajectories and the following points were identified as their major findings:

- Almost all LBP studies confirmed a recurrent episodic or persistent pattern [31]
- Various subgroups have been identified although based on different methods of classification [32–34].
- It was shown that there is a stable pattern across the course of LBP in individuals, and it is very unlikely for someone with severe or long-lasting LBP to become completely pain-free over time and vice versa [33, 35].

However, unless the pain trajectories are properly recorded, it would not be possible to investigate if different trajectories have different prognoses, especially following treatment. Additionally, possible associated triggers can be studied and identified when investigating the trajectories of LBP. This potentially allows the specification of those triggers that exacerbate new episodes of pain and possibly cause different types of trajectories. Also, the knowledge of triggers of episodes of LBP could contribute to the effective prevention and self-management opportunity rather than attempting to ‘cure’ the condition.

Steffens et al., in 2014, sought clinicians’ view on short- and long-term triggering factors of a new episode of LBP and found biomechanical factors as the main contributing causes for both. They, however, have only taken the physiotherapists’ point of view into consideration, not those of the patients’ themselves [36].

In 2015, Steffens et al. conducted a case-crossover study, which looked at exposure to some pre-determined factors that they thought might act as triggers of acute episodes of LBP. They reviewed 999 patients who sought care in a primary care setting. Factors that were considered to contribute to the reoccurrence of a new episode were physical factors such as lifting heavy loads, awkward posture, physical activity, and psychosocial factors like alcohol intake and distraction during activity and fatigue. Exposure was measured 2 h, 24–26 h, and 48–50 h prior to the onset of back pain. All physical triggers were significantly associated with an elevated risk of an acute episode of LBP. Fatigue and distraction among psychosocial triggers were also associated with an increased risk of pain. Nevertheless, in this study, the authors only investigated one acute episode of LBP [37]. A secondary analysis of the same study indicated that the patients are reliably capable of identifying their LBP triggering factors [38].

Another study in 2016 compared patients’ and physiotherapists’ views on triggers for LBP. In this study, even though the category of biomechanical factors was identified as the main trigger by both patients and clinicians, there were some dissimilarities in its subcategory. No new factor was identified. Nonetheless, it seems that patients’ views were their general understanding and not episode-specific [39].

Persuasively, it is likely that the outcome will be more plausible and trustworthy in a study where chronic recurrent LBP sufferers are followed for a more extended period, and triggers are identified for every single episode, since the prognostic association between triggers and trajectories may be recognised. Our literature search failed to reveal any study in which the aforementioned methodological strategies were implemented.

This approach may enable clinical scientists to better understand the course of LBP and its relation to triggers. Also, frequent measurements allow researchers to take the number of episodes and their duration into account to accurately define episodes and non-episodes; something that is still lacking in the literature. Another advantage of repeated measurements is to potentially classify patients into appropriate clinical subgroups based on intensity, frequency, and duration of episodes that may suggest a new approach towards triggering factors, prevention, and prognosis.

### Aims and objectives

The primary aim of the study is to report the triggers of recurring episodes of LBP. The secondary aim is to examine the prognostic association between different triggers and LBP trajectories.

The objectives of this study are:

- To identify participants’ beliefs on the trigger(s) of their episodes of recurring LBP
- To identify self-reported trajectories of recurring LBP
- To identify clinicians’ views on triggers of LBP
- To explore the agreement between patients’ and clinicians’ views on triggers of LBP

## Methods/design

This is a longitudinal, multicentre, Australia-wide observational study of patients with recurrent non-specific LBP. The study has been approved by the Human Research Ethics Committee at the Murdoch University, Perth, Western Australia (project number 2019/034).

### Study flow

**Fig. 1:**
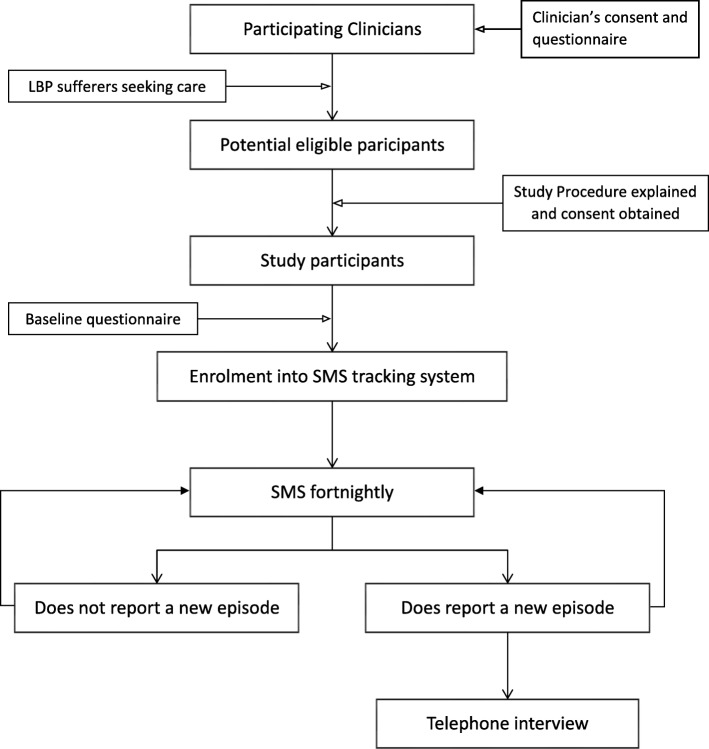
The process of recruitment and follow up

Eligible LBP sufferers who seek care from our participating clinicians are identified for inclusion and will be provided with an information letter and a consent form. Upon signing the consent form, the baseline questionnaire will be given to study participants to complete and be collected by clinicians. Consequently, participants will be enrolled in the SMS tracking system/software via their mobile phone numbers. Figure 1 depicts the process of recruitment and follow-up.

Data in this study will be gathered via a baseline questionnaire, frequent SMS-based questionnairs, telephone interview questionnaires, and clinicians' questionnaires.

After enrolment in the study and registration into the automated SMS system/software, each patient will receive one SMS every 2 weeks to investigate the occurrence of any new episodes of LBP. If the patient reports a new recurrent episode of pain via SMS, they will be contacted via phone by a trained member of the research team, and a telephone interview will be scheduled and performed. If a patient forgets to answer the SMS, which may happen at the beginning, in particular, a member of the research team will contact them by phone and a new explanation of the study procedure/aims will be given. This approach is necessary to ensure high compliance.

### Study participants

Patients with low back pain attending primary care clinics will be encouraged to participate in this study. We intend to recruit patients aged 18 years and older. There is no predilection about the per cent of males and females participating in this study. However, it is anticipated that the proportion will be approximately equal. The sample size is 200 (see below for details) and the study is planned to be conducted Australia wide.

The following criteria are considered for inclusion in the study:

- Patients aged 18 years and over who sought care for pain in the area between the 12th rib and buttock crease
- History of non-specific recurrent low back pain in the past year without leg pain (participants are required to have experienced at least two episodes of LBP with the reported intensity of no less than two on the numerical rating scale (NRS) in addition to a pain-free period in between)
- Mobile (smart) phone possession and ability to use its SMS function
- Ability to understand, read and speak English
- Consent, ability, and willingness to participate for the full duration of the study (52 weeks)

The following criteria will exclude a patient from participating:

- Persistent ongoing LBP, specific LBP such as radiculopathy, disc problems, spinal canal stenosis, fractures, and metastases.
- Secondary and tertiary care seekers
- History of spinal surgery or severe co-morbidities such as osteoarthritis, osteoporosis, uncontrolled diabetes (to be confirmed by our participating clinicians).
- LBP in special populations (e.g. Parkinson’s disease or pregnant women)

### Participating clinicians

Different clinicians and practices (this will include chiropractors, physiotherapists, and GPs) will be approached to help recruit the study sample. Below is the list of potential organisations, practices, and clinicians, who will be contacted and asked for support:

- Murdoch chiropractic alumni (practising anywhere in Australia)
- Murdoch University Chiropractic Clinic (MUCC)
- Australian Osteopathic Association
- Chiropractic Australia
- Australian Chiropractors Association
- The Australian Chiropractic Research Network (ACORN)
- Supervisors at MUCC
- General practitioners within a 10 km radius of Murdoch University
- Physiotherapy clinic at Curtin University of Technology

### Data collection

In this study, data are being collected via different questionnaires:

#### Baseline questionnaire

Sociodemographic data will be collected when the patient consents to participate in the study. This consists of age, sex, height, weight, level of education, level of daily activity, an estimated number of episodes in the preceding year before the baseline, the (visual) trajectory of their pain pattern in the past year [40], and depression and anxiety state. Participants’ anxiety and depression scores will be collected via the 9-item Patient Health Questionnaire (PHQ-9) [41–43] and 7-item generalized anxiety disorder (GAD-7) [44, 45].

#### SMS questionnaire

Once enrolled, each participant receives one SMS fortnightly. Two short, simple questions will be asked in each SMS. Those questions enquire about the number of days with low back pain that had an effect on their daily activities (“troublesome”) in the past two weeks (0–14), and the severity of the pain at its worst (1–10) based on the NRS [46–48]. Patients will receive a reminder or a phone call if they fail to answer a SMS questionnaire.

#### Semi-structured telephone interview

During the telephone interview, details regarding the recent episode of low back pain will be confirmed. These include the location and duration of the recent low back pain experience. An open-ended question will be asked relating to exposure to anything during the 24 h preceding the onset of this episode that the study participants believe could have triggered the current episode. Finally, prior exposure to some well-known and previously documented triggers will be investigated through a structured interview. These are fatigue, manual tasks, moderate and vigorous physical activity, distraction during a task. The open-ended question is asked first so as not to lead the participant.

#### Clinicians’ questionnaire

This short questionnaire contains some basic questions regarding clinician’s name, age, sex, contact details, types of practice/registration, and years in practice (this information is collected for description purposes and determination of diversity of the participating clinicians and study population). In addition, their views on triggers of non-specific low back pain will be solicited.

### Sample size calculation

As Latent Class Analysis (LCA) is not based around hypothesis testing, and there is no formal approach to calculate sample sizes. Critical factors that will affect sample size include prevalence, the sensitivity of items (i.e. class-specific prevalences), and size of classes being fitted in the model. A broadly accurate guide indicated by other researchers uses a minimum of *n* = 200 [49–53]. Three to four classes have been identified as a maximum for the outcomes being explored in this analysis. A sample size of *n* = 200–300 has been based on the optimal minimum sample size required for LCA, allowing for 20% contingency.

A sample size of *n* = 200 has 80% power to detect an odds ratio of 1.35 in adjusted logistic models comparing pain triggers against pain trajectory outcomes (intensity, frequency, length) between four groups (obtained via LCA) over 26-time points, with alpha = 0.05 and beta = 0.2. (G*Power 3.1.7).

### Statistical Analysis

All descriptive summaries of patient characteristics, pain outcomes and pain triggers will include means and standard deviations or medians, interquartile ranges and ranges for continuous data and frequency distributions for categorical data. Data will be grouped according to demographic and other relevant patient factors including gender, age category, BMI category, and amount of physical activity (low, moderate, high) as well as pain pattern trajectories and triggers. All group comparisons will be performed using Chi-square tests for categorical data, and parametric one-way ANOVA or non-parametric Wilcoxon signed rank tests, depending on normality. Normality will be checked using graphical methods and Shapiro-Wilk tests. Data for patients lost to follow-up will be included up until the time of their abandonment.

#### Identifying pain pattern trajectories

Latent Class Analysis (LCA) will be performed in order to identify pain pattern trajectories fortnightly over 12 months based on pain intensity, frequency, and length of episodes. Summaries of patient characteristics for each pain pattern trajectory will be described and compared between trajectories.

Multinomial logistic regression or binary logistic regression using dummy variables will be used to examine patient characteristics as predictors of trajectory membership. Results will be summarised using odds ratios (ORs) and their 95% confidence intervals (CIs).

#### Identifying pain triggers

Pain triggers will be identified from data obtained from open- and close-ended questions in the telephone interview questionnaires. All nominated triggers will be categorised into physical and psychosocial groups.

Summaries of patient characteristics for trigger subgroups will be described and compared between trigger category subgroups such as age category, gender and physical activity categories.

#### Identifying triggers associated with pain pattern trajectories

Generalised linear mixed models with logit links will be used to explore the associations of individual longitudinal pain triggers with pain pattern trajectories. Significant prognostic associations between individual pain triggers and pain pattern trajectories will be identified in adjusted models that will include relevant patients’ and clinicians’ characteristics as covariates. Individual triggers that are significant at *p* < 0.15 in adjusted models will be entered into multivariable models and compared between trajectory groups in the final model. Results will be summarised using odds ratios (ORs) and their 95% confidence intervals (CIs).

Missing values will be accounted for in the mixed model analyses which uses maximum likelihood estimation methods. However, we require a minimum number of 12 Consecutive followups (six months) for the LCA. Stata I/C version 16.0 (StataCorp LLC, College Station TX) will be used to perform data analyses. All analyses will be 2-tailed, and *p*-values < 0.05 will be considered statistically significant. Sensitivity analysis will be performed, comparing patients with full follow-up data against patients with partial follow-up data.

## Discussion and perspectives

Upon completion of this project, trajectories of LBP in participants with recurrent LBP will be identified and reported. A series of newly identified and pre-identified triggers are likely to be found. Any prognostic association between triggers for the different trajectories will be described as will the association between the trajectories and a series of potential moderators on the assumption that they will differ between trajectory subgroups.

Since no treatment has been found to be significantly superior or even better than placebo in the management of non-specific LBP thus far, it may be useful to consider LBP as a chronic condition with concomitant triggers. Therefore, an effective prevention plan and management opportunity might be achieved by dealing with the LBP triggers and trajectories as this may create the possibility of moving the responsibility of care to the patients using education and secondary prevention. It is predicated on the ability of patients being able to recognise the triggers [38] and these triggers to be avoidable.

It is anticipated that outcome yielded from a study in which chronic recurrent LBP sufferers are followed for a longer period, and triggers are identified for every single episode, would offer more reliable preventive measures, management strategies and understanding of prognosis since the association between trajectories and triggers will be recognised.

### Sources of bias

Potential sources of bias and error in this study are:

- Patients’ preconceived ideas of triggers
- Patients’ opinions affected by their treating clinicians’ opinions
- Predominantly chiropractic patients may be different from the broader population of back pain sufferers

Additionally, in this study, we will be comparing different individuals on the basis of their characteristics and also their physical and psychological triggers for their pain outcomes. This leaves some unmeasured factors that could potentially lead to bias.

## Data Availability

Not applicable.

## Abbreviations

LBP: Low back pain
LCA: Latent class analysis
RF: Risk factor
